# Tracking the Continuous Evolutionary Processes of an Endogenous Retrovirus of the Domestic Cat: ERV-DC

**DOI:** 10.3390/v10040179

**Published:** 2018-04-06

**Authors:** Junna Kawasaki, Kazuo Nishigaki

**Affiliations:** Joint Faculty of Veterinary Medicine, Yamaguchi University, 1677-1 Yoshida, Yamaguchi 753-8515, Japan; jrt13mpmuk@gmail.com

**Keywords:** endogenous retrovirus, ERV-DC, feline leukemia virus, feline leukemia virus subgroup D, Refrex-1, retroviral restriction factor, RD-114, domestic cat, *Felis silvestris catus*

## Abstract

An endogenous retrovirus (ERV) is a remnant of an ancient retroviral infection in the host genome. Although most ERVs have lost their viral productivity, a few ERVs retain their replication capacity. In addition, partially inactivated ERVs can present a potential risk to the host via their encoded virulence factors or the generation of novel viruses by viral recombination. ERVs can also eventually acquire a biological function, and this ability has been a driving force of host evolution. Therefore, the presence of an ERV can be harmful or beneficial to the host. Various reports about paleovirology have revealed each event in ERV evolution, but the continuous processes of ERV evolution over millions of years are mainly unknown. A unique ERV family, ERV-DC, is present in the domestic cat (*Felis silvestris* *catus*) genome. ERV-DC proviruses are phylogenetically classified into three genotypes, and the specific characteristics of each genotype have been clarified: their capacity to produce infectious viruses; their recombination with other retroviruses, such as feline leukemia virus or RD-114; and their biological functions as host antiviral factors. In this review, we describe ERV-DC-related phenomena and discuss the continuous changes in the evolution of this ERV in the domestic cat.

## 1. Introduction

Retroviruses can integrate into the host genome through a DNA intermediate in the viral life cycle. Endogenous retroviruses (ERVs) arise from the infection of host germline cells by exogenous retroviruses, which are then transmitted as genetic components to the host’s descendants. Therefore, ERVs are remnants of ancient exogenous retroviruses. Rapid advances in genome projects have shown that ERVs occupy about 4–10% of the human, mouse, and cat genomes [1,2,3,4].

Paleovirology is the study of ancient viruses, including ERVs, to clarify the processes of viral evolution in the long term [5,6,7]. Various paleovirological studies have clarified the events of ancestral virus evolution, including interspecies viral transmission [8,9], virus–host evolutionary arms races [10,11], and the acquisition of host functional genes through ERV domestication [12,13,14,15]. However, the continuous processes of viral evolution over millions of years are largely unknown.

ERV-DC is an endogenous gammaretrovirus of the domestic cat (*Felis silvestris catus*), which is classified into ERV1-3FCa-I by Repbase [16,17,18]. ERV-DC has a simple genomic structure and encodes a Gag–Pol polyprotein and an Env protein in a viral genome of about 8.8 kbp. A unique feature of the ERV-DC family is that the proviruses are phylogenetically classified into three genotypes: genotype I (GI), genotype II (GII), and genotype III (GIII) (Table 1).

**Figure 1:**
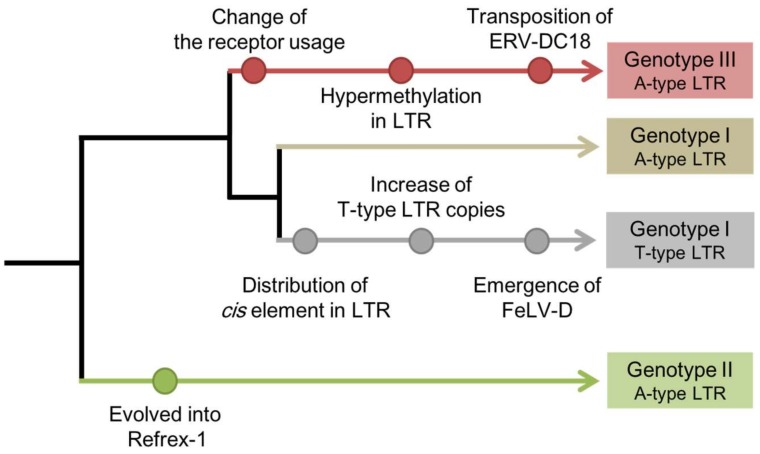
Genotype-specific phenomena in the evolution of ERV-DC. ERV-DC proviruses are phylogenetically classified into three genotypes: genotype I, genotype II, and genotype III. ERV-DC proviruses are also classified into subgroups according to a *cis*-acting element in the LTR: the A-type LTR subgroup has adenine (A) in a *cis*-acting element and has strong promoter activity while the T-type LTR subgroup has thymine (T) in a *cis*-acting element and attenuated promoter activity. Each node point represents an ERV-DC-related phenomenon.

In the last decade, the specific characteristics of each genotype have been clarified (Figure 1). For example, the GI and GIII proviruses can produce replication-competent viruses, and these viruses use different receptors for infection [16,19]. The GI *env* gene has also been transduced into the feline leukemia virus (FeLV), generating a novel interference subgroup: FeLV subgroup D (FeLV-D) [16]. The GII proviruses encode an antiviral factor, Refrex-1, that specifically inhibits ERV-DC GI and FeLV-D infections [20]. In this review, we summarize ERV-DC-related phenomena and discuss the continuous evolutionary processes of ERV-DC.

## 2. Two Distinct Infectious ERV-DC Proviruses with Different Viral Properties

Previous studies have shown that most ERVs are inactivated by the accumulation of mutations after their integration, but a few ERVs retain their replication capacity [21,22,23,24]. The characterization of each proviral genome of ERV-DC indicated that most of these proviruses lose their infectivity. However, ERV-DC14 belonging to GI, ERV-DC10, and ERV-DC18 belonging to GIII can produce infectious viruses [16,19], and representative morphologically C-type retrovirus-like particles have been observed with transmission electron microscopy in cells infected with these proviruses.

Here, we describe the loci that retain the virus replication ability for each genotype. Among the GI proviruses, a viral replication ability has only been confirmed in ERV-DC14 [19]. A fluorescence in situ hybridization (FISH) analysis mapped ERV-DC14 to the feline chromosome C1q32. A survey of the insertion polymorphisms of ERV-DC loci indicated that only 2.5% of the Japanese domestic cats tested carried ERV-DC14, so ERV-DC14 is present at a low frequency in the host population [16]. Interestingly, although the ERV-DC8 provirus has an intact genetic structure very similar to that of ERV-DC14, no viral productivity has been observed. Therefore, a comparison of ERV-DC14 and ERV-DC8 may identify the functional domain responsible for viral replication.

ERV-DC10 and ERV-DC18, which belong to GIII, differ at only one base in a primer-binding site in the full-length proviral genome [16]. A FISH analysis mapped ERV-DC10 and ERV-DC18 to the feline chromosomes C1q12-21 and D4q14, respectively. Therefore, two different loci in GIII produce infectious viruses. A survey of ERV-DC insertion polymorphisms showed that 37.7% of the Japanese domestic cats tested carried ERV-DC10, but ERV-DC18 was detected in only one sample (cat ON-T). Further investigation detected ERV-DC18 in two cats related to cat ON-T, so ERV-DC18 is considered to have been recently endogenized by infection or transposition of ERV-DC GIII. Thus, these results suggested that GIII contains active proviruses that still retain viral replication potential or transposition activity.

Although both GI and GIII contain proviruses with the ability to produce infectious viruses, their viral properties differ in two respects: their receptor usage and viral titers. An interference assay indicated that GI and GIII use different receptors for infection [16,19]. GI can infect cell lines derived from various mammals, including the domestic cat, human, monkey, dog, and cow. In contrast, GIII infects limited cell lines, including those of human and dog. Interestingly, GIII is unable to infect cat cells, such as AH927 fibroblasts and G355 astrocytes, in which the GIII provirus is integrated. Therefore, GI is a nonecotropic virus, whereas GIII is a xenotropic virus. Identifying the receptors used by GI and GIII for infection has clarified the differences in their infectious tropism [25,26,27,28,29,30].

The viral titer of the ERV-DC14 (GI) is lower than those of the ERV-DC10 and ERV-DC18 (GIII). Therefore, ERV-DC14 fails to establish a persistent infection in human cultured cells, whereas ERV-DC10 and -DC18 can persistently infect human cells [19]. (The detailed differences in their viral titers are described in a later chapter: “Genotype-specific strategies of ERV-DCs in transcriptional regulation”.)

## 3. Transduction of ERV-DC GI *env* Gene into Feline Leukemia Virus ( FeLV)

FeLV is a pathogenic gammaretrovirus of the domestic cat that causes various pathologies in the host, including proliferative diseases and immunosuppressive diseases, with high mortality rates [31,32]. The FeLV subgroups are classified according to their infectious tropism, based on differences in the receptors they use for infection [30,33,34,35,36,37,38,39]. FeLV subgroup A is the commonest subgroup, and is transmitted horizontally between host individuals through grooming, sharing of food, or biting.

**Figure 2:**
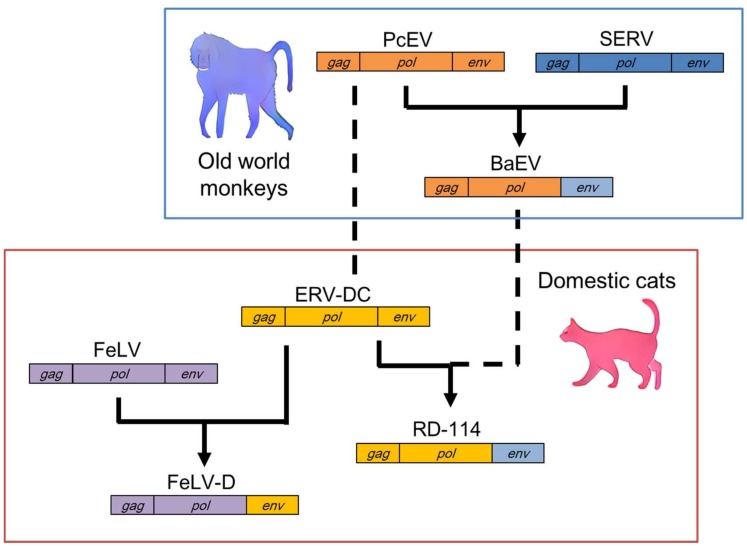
Recombination events between ERV-DC and other retroviruses. Feline leukemia virus subgroup D (FeLV-D) was generated from the FeLV *gag-pol* genes and the ERV-DC genotype I *env* gene. Baboon endogenous retrovirus (BaEV) was generated from the *gag-pol* genes of *Papio cynocephalus* endogenous retrovirus (PcEV) and the *env* gene of simian endogenous retrovirus (SERV). RD-114 was generated from the ERV-DC *gag-pol* genes and the *env* gene of BaEV. Dotted lines indicate interspecies transmission among old world monkeys and cats.

The discovery of a novel recombinant FeLV genome that had captured viral fragments of ERV-DC prompted the characterization of ERV-DC [16,40]. The recombinant FeLV was designated “FeLV-D” because it belongs to a different interference group from the known FeLV subgroup. Both a genetic analysis and interference experiments showed that FeLV-D had acquired the *env* gene of ERV-DC GI [16,20,39]. Therefore, ERV-DC GI has contributed a new feature to a modern virus (Figure 2).

FeLV-D was identified in four cats, including two cats that were related. Clinical information on the FeLV-D-infected cats showed that three of the cats had hematopoietic tumors, including lymphoma and leukemia [41]. Further investigations, such as an ingestion experiment in laboratory animals, are required to analyze the virulence of FeLV-D in more detail.

In the FeLV-D *env* genes detected in the four infected cats, the 3′ recombination junctions in the transmembrane unit (TM) differed from each other, although the full-length surface units (SUs) of all the clones were replaced with that of ERV-DC GI. This suggests that FeLV-D was generated de novo in each cat. It has been reported that in other subgroups of FeLV, slight differences in genetic structure lead to changes in infection tropism or pathogenicity [30,42]. Therefore, it is possible that the differences in the viral properties of the FeLV-D clones are caused by variations of the recombination junction.

## 4. ERV-DC GII Encodes the Host Antiviral Factor Refrex-1

In rare cases, ERV-derived sequences have gained an activity that contributes to the maintenance of the host physiology, and this phenomenon is called “ERV domestication”. ERV domestication is exemplified by antiviral factors [14,21], placenta formation ability [43], myoblast fusion ability [44], or an mRNA transporter in the nervous system [45,46]. These reports suggest that ERV domestication has contributed dramatically to the evolution of the host.

During the characterization of FeLV-D, we unexpectedly detected an unknown antiviral factor directed against FeLV-D and ERV-DC GI in the supernatant of 3201 feline T cells [20]. We designated the antiviral factor “restriction for feline retrovirus X (Refrex-1)”. To determine the gene that encodes Refrex-1, we screened soluble molecules associated with ERV-DC in a cDNA library synthesized from transcripts in 3201 cells, and two suspected clones were identified. Further experimentation revealed that these two clones inhibited FeLV-D and ERV-DC GI infections in a dose-dependent manner.

**Figure 3:**
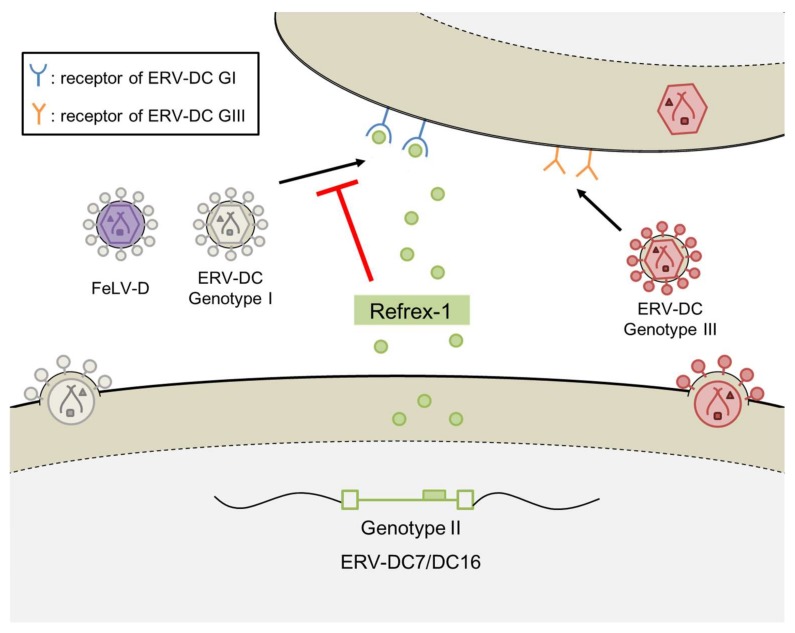
Defense mechanism of Refrex-1.

Interestingly, the genetic structures of the two clones were identical to the *env* genes of ERV-DC7 and ERV-DC16, and experiments using cells transfected with ERV-DC7 or ERV-DC16 confirmed the same antiviral activity as Refrex-1. These results indicate that ERV-DC7 and ERV-DC16, belonging to ERV-DC GII, have been domesticated as Refrex-1 to protect the host from viral infection. Further experiments showed that the defense mechanism of Refrex-1 involves the extracellular secretion of Refrex-1, which interferes with the receptors for FeLV-D and ERV-DC GI infection (Figure 3). In contrast to FeLV-D and ERV-DC GI infections, Refrex-1 does not block ERV-DC GIII infections, presumably because the infectious receptors used by ERV-DC GIII differ from those used by ERV-DC GI and FeLV-D.

The *env* genes of both ERV-DC7 and ERV-DC16 are truncated by stop codons at amino acids 252 and 298, respectively, in the proline-rich region (PRR). One of the unique properties of Refrex-1 is that it consists of a truncated Env protein including an SU domain and signal peptide [14]. These structures, which lack a TM, appear to express efficient antiviral activity for the following two reasons. First, Refrex-1 can be secreted from cells without remaining on the cell membrane, and causes extracellular receptor interference. The infection efficiencies of FeLV-D and ERV-DC GI were significantly reduced in cells when the supernatants of a variety of cultured feline cells were added. Second, Refrex-1 does not include the immunosuppressive domain (ISD). The ISD of ERV-DC closely resembles that of other gammaretroviruses whose immunosuppressive activities have been demonstrated [47,48,49]. Therefore, it is conceivable that structures lacking the ISD can protect the host against viral infection without impairing its immunity.

The proportion of cats shown to carry ERV-DC7 or ERV-DC16 indicates that both loci are fixed in the cat genome [16]. Therefore, the antiviral activity of Refrex-1 may have contributed to the survival of the host during feline evolution. A genotype-specific expression analysis using cat organs showed high expression levels of GII in most organs, especially in the peripheral blood [19]. However, many aspects of Refrex-1 functions in vivo remain unknown, including the extent to which Refrex-1 can inhibit infection by FeLV-D or ERV-DC GI, and whether ERV-DC7 or ERV-DC16 can act as Refrex-1.

## 5. Refrex-1 Is under Robust Control by Accumulated Inactivation Mechanisms

Because it was unclear how ERV-DC7 and ERV-DC16 evolved into Refrex-1, we investigated the process of Refrex-1 evolution using the reconstructed full-length *env* genes of ERV-DC7 and ERV-DC16 (ERV-DC7fl and ERV-DC16fl, respectively) [50]. An infectivity analysis of ERV-DC7fl and ERV-DC16fl revealed that they are unable to produce viruses because the cleavage between SU and TM is disrupted. Therefore, Refrex-1 is strictly controlled by multiple inactivation steps, including stop codons in the PRR and cleavage failure.

Additional analyses provided interesting data on ERV-DC7fl. First, the reconstructed *env* gene showed antiviral activity, like Refrex-1, even when ERV-DC7fl appeared to remain on the cell surface. Second, a comparative analysis of the ERV-DC7 alleles showed that purifying selection is active not only in the domain that currently functions as Refrex-1, but also in the entire *env* gene. Third, a sequence comparison revealed recombination phenomena among the alleles of the ERV-DC7 *env* gene.

These results prompted the following hypothesis about the processes underlying the evolution of ERV-DC7 into Refrex-1. Before evolving to the current Refrex-1, intermediate alleles of the ERV-DC7 *env* gene existed. For example, one was inactivated by a stop codon in the PRR, and another was inactivated by cleavage dysfunction. Among these intermediates, several alleles appear to have acquired antiviral activity. The recombination phenomena among the ERV-DC7 alleles suggest that the stricter control of viral activity, which is a prerequisite for ERV domestication, has been gradually achieved by the accumulation of inactivation steps during recombination. To test this hypothesis, ERV-DC7 must be detected in other species of the genus *Felis* and compared with that in the domestic cat.

## 6. Genotype-Specific Transcriptional Regulation Strategies in ERV-DCs

As described above, the characteristics of the ERV-DC proviruses seem to be associated with their genotypes. ERV-DC GI and GIII include replication-competent proviruses, and the infectious activities of ERV-DC10 and -DC18 (GIII) are much stronger than those of ERV-DC14 (GI) [16,19]. ERV-DC7 and -DC16 encode the antiviral factor Refrex-1 [20]. Because the three ERV-DC genotypes show distinct characteristics, we speculated that these genotypes are controlled in distinct ways. To clarify this point, we investigated the transcription patterns and regulatory mechanisms of ERV-DCs at genotype- and locus-level resolution.

The ERV-DC genotype-specific expression profiles were characterized in various feline organs [19]. The expression of GII was high in almost all tissues, whereas that of GI and GIII was extremely low. These results appear to be consistent with the fact that GII contributes to the host’s viral defenses as Refrex-1, and that GI and GIII include proviruses capable of producing infectious viruses.

To clarify the mechanism underlying the differences in their transcriptional activities, we investigated the regulatory mechanisms involved. First, the methylation status of the CpG islands in the 5′ long terminal repeat (5′LTR) of each ERV-DC provirus was analyzed. Genotype-specific methylation levels were identified: the GIII 5′LTR is strongly methylated along its full length, whereas the GI 5′LTR is partially methylated downstream from the TATA box. By contrast, the GII 5′LTR is negligibly methylated.

Secondly, we analyzed the basal promoter activity of the 5′LTR in each provirus. Promoter activity was confirmed in the GII, GIII, and several GI proviruses (ERV-DC19 and ERV-DC2), but not in other GI proviruses (ERV-DC1, -DC3, -DC4, -DC8, -DC14, and -DC17). Further investigation with a chimeric LTR constructed from ERV-DC19 and ERV-DC8 identified the nucleotide substitution responsible for the difference in the basal promoter activities. Although ERV-DC14 is incapable of persistent infection, ERV-DC14TA, in which the substitution determining the promoter activity was repaired, established persistent infections like those of ERV-DC10 and ERV-DC18.

These results indicate that the observed genotype-specific transcriptional regulation is attributable to two distinct mechanisms in the 5′LTR: the transcriptional activity of GI is controlled by partial CpG methylation and a point mutation in a *cis*-acting element, whereas that of GIII is restricted by strong CpG methylation. In contrast to GI and GIII, there are few restrictions in GII expression.

The LTRs of ERV-DC were classified into two subgroups based on the identification of the *cis*-acting element: the A-type LTR subgroup, with high promoter activity; and the T-type LTR subgroup, with low activity (Figure 1). Interestingly, a comprehensive search of the domestic cat genome indicated that the copy number of the T-type LTR is significantly higher than that of the A-type LTR, despite the low promoter activity of the T-type LTR. One hypothesis explaining this phenomenon is that ERV-DCs with T-type LTRs have escaped from negative selective pressure because they have lost their strong promoter activity, which could reduce the fitness of the host. Another hypothesis is that the T-type LTR has been optimized to the transcriptional environment of germ cells in order to replicate efficiently in those cells [51]. However, further analyses, such as the identification of the transcription factors that bind the A-type and T-type LTRs, are required to test these hypotheses.

## 7. Multiple Recombination Events between ERV-DC and RD-114

RD-114 was first isolated from fetal kittens that had ingested human rhabdomyosarcoma cells, and subsequent studies showed that RD-114 is a domestic cat ERV [52]. The identification of ERV-DC revealed that RD-114 is a chimeric virus composed of the *gag*-*pol* genes of ERV-DC and the *env* gene of a baboon endogenous retrovirus (BaEV) (Figure 2) [16,18]. Other studies have shown that BaEV is also a chimeric virus composed of the *gag*-*pol* genes of a *Papio cynocephalus* endogenous retrovirus (PcEV) and the *env* gene of a simian endogenous retrovirus (SERV) [53,54,55]. Therefore, RD-114 was generated by at least two recombination events: the transduction of SERV *env* into PcEV and the transduction of BaEV *env* into ERV-DC [56]. Interestingly, PcEV appears to be a counterpart of ERV-DC in the baboon, because these genomes share approximately 70% similarity. These results suggest that multiple interspecies transmissions have occurred among old world monkeys and cats, with several subsequent recombination events among the transmitted viruses.

Although the existence of both ERV-DC and RD-114 has not been confirmed in the chromosomal DNA of the Tsushima wild cat (*Prionailurus bengalensis euptilurus*), it is predicted that a recombination event between ERV-DC and BaEV occurred about 6.2–9.3 million years ago (mya), when the genus *Felis* and the genus *Prionailurus* seem to have separated [16,57]. However, it is still unclear how ERV-DC and RD-114 coexisted within the same host. Therefore, we analyzed the details of these recombination events using ERV-DC, RD-114, and RD114-virus-related sequences (RDRSs) [58,59].

**Figure 4:**
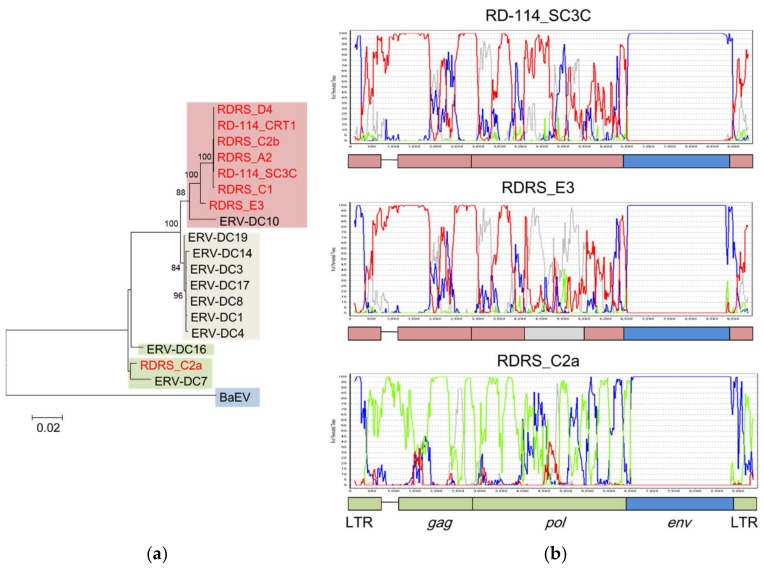
Repeated transduction of ERV-DC into RD-114 in independent events. (**a**) Phylogenetic tree based on the nucleotide sequences of the *pol* genes of ERV-DC, RD-114, and RD114-virus-related sequences (RDRSs). RD-114 and RDRS are shown in red letters. The sequences are shown in the following colors, according to clade: ERV-DC genotype I (GI) clade: gray; ERV-DC genotype II (GII) clade: green; ERV-DC genotype III (GIII) clade: red; and baboon endogenous retrovirus (BaEV) clade: blue. (**b**) Recombination analysis of RD-114_SC3C, RDRS_E3, and RDRS_C2a with bootscanning, using the nucleotide sequences of ERV-DC GI, GII, GIII, and BaEV. The vertical axis represents the percentage of permuted trees and the horizontal axis represents the alignment position. Each line is colored as follows: ERV-DC GI, gray; ERV-DC GII, green; ERV-DC GIII, red; and BaEV, blue. The bootscanning analysis was conducted with Simplot [62]. Sequences for the analysis were extracted with a window size of 200 bp and a step size of 20 bp, and each phylogenetic analysis was repeated 100 times with the neighbor-joining method based on the Kimura 2-parameter model [63]. The accession numbers of the nucleotide sequences are: ERV-DC: AB674439–AB674452, AB807599, and AB807600; RD-114_CRT1: AB559882; RD-114_SC3C: NC_009889; RDRSs: LC005744–LC005749; and BaEV: D10032.

A phylogenetic analysis suggested that RD-114 (RD-114_CRT1 and RD-114_SC3C) and most RDRSs (RDRS_A2, RDRS_C2b, RDRS_D4, RDRS_C1, and RDRS_E3) were generated by the transduction of ERV-DC GIII, whereas only RDRS_C2a is composed of the ERV-DC GII sequence (Figure 4A). The estimated time of integration, based a comparison of their LTRs, indicated that the age of ERV-DC7 (belonging to GII) is around 2.8 mya and that of RDRS_C2a is around 1.6 mya [16,59]. However, we speculated that almost all ERV-DC GI and GIII and other RDRSs were integrated into the feline genome quite recently, at less than 0.2 mya. The recombination analysis also showed that the *gag* and *pol* genes of RD-114_SC3C and RDRS_C2a derived from ERV-DC GIII and GII, respectively, and these results are consistent with those of the phylogenetic analysis (Figure 4B). In contrast, the recombination analysis indicated that a part of the *pol* gene of RDRS_E3 is derived from ERV-DC GI. Therefore, these results suggest that RD-114 has repeatedly recombined with each genotype of ERV-DC over a prolonged period.

In this review, the occurrence of multiple recombination events between ERV-DC and RD-114 has been suggested. However, there is little insight into where and when these recombination events occurred. Furthermore, the interspecies transmission of the virus between old world monkeys and domestic cats, which underpins the occurrence of recombination, is largely unclear. To clarify these issues, it will be necessary to combine a comprehensive viral sequence analysis and a host species analysis from both evolutionary and ecological perspectives, as has been reported in other studies of BaEV and PcEV evolution [54,55,60,61].

## 8. Evolutionary Scenario of ERV-DC

The endogenization of a virus leads to dynamic changes in the host genome, but it is unclear whether the results are detrimental or beneficial to the host. In this review, the main characteristics specific to each ERV-DC genotype have been described. ERV-DC GI and GIII contain loci that confer viral replicative or translocation abilities, which may threaten the host’s life [16,19]. Moreover, the emergence of FeLV-D, a viral recombinant of ERV-DC GI and FeLV, may pose a new hazard to domestic cats [16]. However, in contrast, the presence of ERV-DC GII has benefited the survival of the host population by introducing the antiviral factor Refrex-1, which has prevented the expansion of ancient ERV-DC infections and modern FeLV-D infections [20].

The rearrangement of ERV-DC-related phenomena is analogous to the continuous changes occurring in the evolution of the ERV-DC family, and the evolutionary scenario suggests that the appearance of Refrex-1 introduced a branch point in the evolution of ERV-DC (Figure 1). Therefore, to escape the effects of Refrex-1, some ERV-DCs appear to have changed the receptors they use for infection, and became the current GIII viruses. Similarly, the evolution of RD-114 may have also circumvented the antiviral activity of Refrex-1 by altering the *env* gene.

The various ERV-DC genotypes imply that the three genotypes were endogenized independently in terms of geography or era, but the evolutionary origin of ERV-DC is largely unknown. Further analysis of ERV-DC in other species of the genus *Felis* may clarify the evolutionary processes of ERV-DC.

## 9. Concluding Remarks

The rearrangement of ERV-DC-related phenomena suggests a scenario of ERV-DC evolution and raises new questions. In particular, the following factors must be clarified in more detail: the viral pathogenicity of ERV-DC and FeLV-D, the domestication of ERV-DC-derived sequences, and the long-term processes of ERV-DC evolution.

To address the first question, the potential viral pathogenicity of ERV-DC proviruses belonging to GI and GIII must be investigated, together with the novel viral pathogenicity of FeLV-D. Analyzing the pathogenicity of these viruses is important not only in paleovirology, but also in risk management to control the emerging and reemerging viruses.

Other studies have reported the appearance of new viruses with the reassortment of viral genes [64,65]. Because most ERV-DC proviruses retain their potential genetic structure, we must also consider the possibility of viral gene reassortment (Table 1).

Previous studies have also indicated that abnormal ERV expression may be associated with diseases such as cancer [66] or autoimmune diseases [67], although the relationship between ERV-DC expression and disease remains to be investigated. For this purpose, it will be necessary to analyze expression data from both normal and diseased tissues.

To address the second question, the function of Refrex-1 must be demonstrated in vivo. We believe that Refrex-1 may have a physiological function as a secreted protein other than its antiviral activity.

Furthermore, because most ERV-DC proviruses have an intact open reading frame (ORF), we must consider whether other ERV-DC proviruses have acquired new biological functions. In particular, ERV-DC6, which seems to be fixed within the domestic cat population, retains an intact ORF in the *env* gene. Therefore, it is possible that ERV-DC6 has gained a function as a host gene.

A comparison of ERV-DC across the genus *Felis* should be effective in addressing the third question. (The details are described in the chapter “Evolutionary scenario of ERV-DC”.)

Further exploration of these factors will clarify the continuous evolution of ERV-DC on a finer time scale. Studies of the continuous evolution of ERV-DC will extend our paleovirological understanding, including the evolutionary history of retroviruses over long time periods and the roles of endogenous viruses in host evolution. 

## Figures and Tables

**Table 1:** Characteristics of each domestic cat endogenous retrovirus (ERV-DC) provirus.

Genotype	Provirus	Intact ORF ^1^	Viral Productivity ^2^	Functional Gene
Genotype I	ERV-DC1	−	−	
	ERV-DC2	*env*	?	
	ERV-DC3	−	−	
	ERV-DC4	*gag-pol*	−	
	ERV-DC8	*gag-pol*, *env*	−	
	ERV-DC14	*gag-pol*, *env*	+	
	ERV-DC17	*gag*, *env*	−	
	ERV-DC19	*env*	−	
Genotype II	ERV-DC7	SU ^3^	−	Refrex-1
	ERV-DC16	*gag*, SU	−	Refrex-1
Genotype III	ERV-DC6	*env*	?	
	ERV-DC10	*gag-pol*, *env*	+	
	ERV-DC18	*gag-pol*, *env*	+	

^1^ Open reading frame (ORF); ^2^ + means replication-competent and − means replication-incompetent. Because ERV-DC2 and ERV-DC6 have been identified as partial genomes, their virus productivity is still unknown (?); ^3^ surface unit in the *env* gene (SU).

## References

[1] Mager D.L., Stoye J.P. (2015). Mammalian endogenous retroviruses. Microbiol. Spectr..

[2] International Human Genome Sequencing Consortium (2001). Initial sequencing and analysis of the human genome. Nature.

[3] Mouse Genome Sequencing Consortium (2002). Initial sequencing and comparative analysis of the mouse genome. Nature.

[4] Pontius J.U., Mullikin J.C., Smith D.R., Lindblad-Toh K., Gnerre S., Clamp M., Chang J., Stephens R., Neelam B., Volfovsky N. (2007). Initial sequence and comparative analysis of the cat genome. Genome Res..

[5] Emerman M., Malik H.S. (2010). Paleovirology—Modern consequences of ancient viruses. PLoS Biol..

[6] Magiorkinis G., Belshaw R., Katzourakis A. (2013). “There and back again”: Revisiting the pathophysiological roles of human endogenous retroviruses in the post-genomic era. Philos. Trans. R. Soc. Lond. B.

[7] Aiewsakun P., Katzourakis A. (2015). Endogenous viruses: Connecting recent and ancient viral evolution. Virology.

[8] Diehl W.E., Patel N., Halm K., Johnson W.E. (2016). Tracking interspecies transmission and long-term evolution of an ancient retrovirus using the genomes of modern mammals. eLife.

[9] Zhuo X., Feschotte C. (2015). Cross-species transmission and differential fate of an endogenous retrovirus in three mammal lineages. PLoS Pathog..

[10] Imbeault M., Helleboid P.-Y., Trono D. (2017). KRAB zinc-finger proteins contribute to the evolution of gene regulatory networks. Nature.

[11] Jern P., Coffin J.M. (2008). Host-retrovirus arms race: Trimming the budget. Cell Host Microbe.

[12] Garcia-Perez J.L., Widmann T.J., Adams I.R. (2016). The impact of transposable elements on mammalian development. Development.

[13] Chuong E.B., Elde N.C., City S.L. (2016). Regulatory evolution of innate immunity through co-option of endogenous retroviruses. Science.

[14] Malfavon-Borja R., Feschotte C. (2015). Fighting fire with fire: Endogenous retrovirus envelopes as restriction factors. J. Virol..

[15] Buzdin A.A., Prassolov V., Garazha A.V. (2017). Friends-Enemies: Endogenous Retroviruses Are Major Transcriptional Regulators of Human DNA. Front. Chem..

[16] Anai Y., Ochi H., Watanabe S., Nakagawa S., Kawamura M., Gojobori T., Nishigaki K. (2012). Infectious endogenous retroviruses in cats and emergence of recombinant viruses. J. Virol..

[17] Beyer W., Mohring R., Drescher B., Notzel U., Rosenthal S. (1987). Molecular cloning of an endogenous cat retroviral element (ECE 1)—A recombinant between RD-114 and FeLV-related sequences. Arch. Virol..

[18] Van der Kuyl A.C., Dekker J.T., Goudsmit J. (1999). Discovery of a new endogenous type C retrovirus (FcEV) in cats: Evidence for RD-114 being an FcEV(Gag-Pol)/baboon endogenous virus BaEV(Env) recombinant. J. Virol..

[19] Kuse K., Ito J., Miyake A., Kawasaki J., Watanabe S., Makundi I., Ngo M.H., Otoi T., Nishigaki K. (2016). Existence of two distinct infectious endogenous retroviruses in domestic cats and their different strategies for adaptation to transcriptional regulation. J. Virol..

[20] Ito J., Watanabe S., Hiratsuka T., Kuse K., Odahara Y., Ochi H., Kawamura M., Nishigaki K. (2013). Refrex-1, a soluble restriction factor against feline endogenous and exogenous retroviruses. J. Virol..

[21] Kozak C. (2014). Origins of the Endogenous and Infectious Laboratory Mouse Gammaretroviruses. Viruses.

[22] Tarlinton R.E., Meers J., Young P.R. (2006). Retroviral invasion of the koala genome. Nature.

[23] Le Tissier P., Stoye J.P., Takeuchi Y., Patience C., Weiss R.A. (1997). Two sets of human-tropic pig retrovirus. Nature.

[24] Aaronson S.A., Tronick S.R., Stephenson J.R. (1976). Endogenous type C RNA virus of Odocoileus hemionus, a mammalian species of New World origin. Cell.

[25] Albritton L.M., Tseng L., Scadden D., Cunningham J.M. (1989). A putative murine ecotropic retrovirus receptor gene encodes a multiple membrane-spanning protein and confers susceptibility to virus infection. Cell.

[26] Tailor C.S., Nouri A., Lee C.G., Kozak C., Kabat D. (1999). Cloning and characterization of a cell surface receptor for xenotropic and polytropic murine leukemia viruses. Proc. Natl. Acad. Sci. USA.

[27] Battini J.L., Rasko J.E., Miller AD. (1999). A human cell-surface receptor for xenotropic and polytropic murine leukemia viruses: Possible role in G protein-coupled signal transduction. Proc. Natl. Acad. Sci. USA.

[28] Yang Y.-L., Guo L., Xu S., Holland C.A., Kitamura T., Hunter K., Cunningham J.M. (1999). Receptors for polytropic and xenotropic mouse leukaemia viruses encoded by a single gene at Rmc1. Nat. Genet..

[29] Riedel N., Hoover E.A., Dornsife R.E., Mullins J.I. (1988). Pathogenic and host range determinants of the feline aplastic anemia retrovirus. Proc. Natl. Acad. Sci. USA.

[30] Shalev Z., Duffy S.P., Adema K.W., Prasad R., Hussain N., Willett B.J., Tailor C.S. (2009). Identification of a feline leukemia virus variant that can use THTR1, FLVCR1, and FLVCR2 for infection. J. Virol..

[31] Hartmann K. (2011). Clinical aspects of feline immunodeficiency and feline leukemia virus infection. Vet. Immunol. Immunopathol..

[32] Bolin L.L., Levy L.S. (2011). Viral determinants of FeLV infection and pathogenesis: Lessons learned from analysis of a natural cohort. Viruses.

[33] Sarma P.S., Log T. (1973). Subgroup classification of feline leukemia and sarcoma viruses by viral interference and neutralization tests. Virology.

[34] Mendoza R., Anderson M.M., Overbaugh J. (2006). A putative thiamine transport protein is a receptor for feline leukemia virus subgroup A.

[35] Takeuchi Y., Vile R.G., Simpson G.U.Y., Hara B.O., Collins M.K.L., Weiss R.A. (1992). Feline leukemia virus subgroup B uses the same cell surface receptor as gibbon ape leukemia virus. J. Virol..

[36] Tailor C.S., Willett B.J., Kabat D. (1999). A putative cell surface receptor for anemia-inducing feline leukemia virus subgroup C is a member of a transporter superfamily. J. Virol..

[37] Quigley J.G., Burns C.C., Anderson M.M., Lynch E.D., Sabo K.M., Overbaugh J., Abkowitz J.L. (2000). Cloning of the cellular receptor for feline leukemia virus subgroup C (FeLV-C), a retrovirus that induces red cell aplasia. Blood.

[38] Anderson M.M., Lauring A.S., Burns C.C. (2000). Identification of a cellular cofactor required for infection by feline leukemia virus.

[39] Miyake A., Watanabe S., Hiratsuka T., Ito J., Ngo M.H., Makundi I., Kawasaki J., Endo Y., Tsujimoto H., Nishigaki K. (2016). Novel feline leukemia virus interference group based on the env gene. J. Virol..

[40] Watanabe S., Kawamura M., Odahara Y., Anai Y., Ochi H., Nakagawa S., Endo Y., Tsujimoto H., Nishigaki K. (2013). Phylogenetic and structural diversity in the feline leukemia virus *env* gene. PLoS ONE.

[41] Ngo M.H., Nishigaki K. Pathological analysis of Feline Leukemia Virus subgroup D (FeLV-D)-infected cats.

[42] Boomer S., Eiden M., Burns C.C. (1997). Three distinct envelope domains, variably present in subgroup B feline leukemia virus recombinants, mediate Pit1 and Pit2 receptor recognition. J. Virol..

[43] Lavialle C., Cornelis G., Dupressoir A., Esnault C., Heidmann O., Vernochet C., Heidmann T. (2013). Paleovirology of “syncytins”, retroviral *env* genes exapted for a role in placentation. Philos. Trans. R. Soc. B.

[44] Redelsperger F., Raddi N., Bacquin A., Vernochet C., Mariot V., Gache V., Blanchard-Gutton N., Charrin S., Tiret L., Dumonceaux J. (2016). Genetic evidence that captured retroviral envelope syncytins contribute to myoblast fusion and muscle sexual dimorphism in mice. PLOS Genet..

[45] Pastuzyn E.D., Day C.E., Kearns R.B., Kyrke-Smith M., Taibi A. V, McCormick J., Yoder N., Belnap D.M., Erlendsson S., Morado D.R. (2018). The Neuronal Gene *Arc* Encodes a Repurposed Retrotransposon Gag Protein that Mediates Intercellular RNA Transfer. Cell.

[46] Ashley J., Cordy B., Lucia D., Fradkin L.G., Budnik V., Thomson T. (2018). Retrovirus-like Gag protein Arc1 binds RNA and traffics across synaptic boutons. Cell.

[47] Cianciolo G.J., Kipnis R.J., Snyderman R. (1984). Similarity between p15E of murine and feline leukaemia viruses and p21 of HTLV. Nature.

[48] Mang R., Maas J., Chen X., Goudsmit J., van der Kuyl A.C. (2001). Identification of a novel type C porcine endogenous retrovirus: Evidence that copy number of endogenous retroviruses increases during host inbreeding. J. Gen. Virol..

[49] Schlecht-Louf G., Mangeney M., El-Garch H., Lacombe V., Poulet H., Heidmann T. (2014). A targeted mutation within the feline leukemia virus (FeLV) envelope protein immunosuppressive domain to improve a canarypox virus-vectored FeLV vaccine. J. Virol..

[50] Ito J., Baba T., Kawasaki J., Nishigaki K. (2015). Ancestral mutations acquired in Refrex-1, a restriction factor against feline retroviruses, during its cooption and domestication. J. Virol..

[51] Ito J., Sugimoto R., Nakaoka H., Yamada S., Kimura T., Hayano T., Inoue I. (2017). Systematic identification and characterization of regulatory elements derived from human endogenous retroviruses. PLoS Genet..

[52] McAllister R.M., Nicolson M., Gardner M.B., Rongey R.W., Rasheed S., Sarma P.S., Huebner R.J., Hatanaka M., Oroszlan S., Gilden R.V. (1972). C-type virus released from cultured human rhabdomyosarcoma cells. Nat. New Biol..

[53] Van der Kuyl A.C., Dekker J.T., Goudsmit J. (1995). Full-length proviruses of baboon endogenous virus (BaEV) and dispersed BaEV Reverse transcriptase retroelements in the genome of baboon species. J. Virol..

[54] Van der Kuyl A.C., Mang R., Dekker J.T., Goudsmit J. (1997). Complete nucleotide sequence of simian endogenous type D retrovirus with intact genome organization: Evidence for ancestry to simian retrovirus and baboon endogenous virus. J. Virol..

[55] Mang R., Goudsmit J., van der Kuyl A.C. (1999). Novel endogenous type C retrovirus in baboons: Complete sequence, providing evidence for baboon endogenous virus *gag-pol* ancestry. J. Virol..

[56] Kim F.J., Battini J.L., Manel N., Sitbon M. (2004). Emergence of vertebrate retroviruses and envelope capture. Virology.

[57] Johnson W.E. (2006). The late Miocene radiation of modern Felidae: A genetic assessment. Science.

[58] Yoshikawa R., Sato E., Igarashi T., Miyazawa T. (2010). Characterization of RD-114 virus isolated from a commercial canine vaccine manufactured using CRFK cells. J. Clin. Microbiol..

[59] Shimode S., Nakagawa S., Miyazawa T. (2015). Multiple invasions of an infectious retrovirus in cat genomes. Sci. Rep..

[60] Mang R., Maas J., van Der Kuyl A.C., Goudsmit J. (2000). Papio cynocephalus endogenous retrovirus among old world monkeys: Evidence for coevolution and ancient cross-species transmissions. J. Virol..

[61] Van der Kuyl A.C., Dekker J.T., Goudsmit J. (1995). Distribution of baboon endogenous virus among species of african monkeys suggests multiple ancient cross-species transmissions in shared habitats. J. Virol..

[62] Lole K.S., Bollinger R.C., Paranjape R.S., Gadkari D., Kulkarni S.S., Novak N.G., Ingersoll R., Sheppard H.W., Ray S.C. (1999). Full-length human immunodeficiency virus type 1 genomes from subtype C-infected seroconverters in India, with evidence of intersubtype recombination. J. Virol..

[63] Kimura M. (1980). A simple method for estimating evolutionary rates of base substitutions through comparative studies of nucleotide sequences. J. Mol. Evol..

[64] Paprotka T., Delviks-Frankenberry K.A., Cingöz O., Martinez A., Kung H.J., Tepper C.G., Hu W.S., Fivash M.J., Coffin J.M., Pathak V.K. (2011). Recombinant origin of the retrovirus XMRV. Science.

[65] Young G.R., Eksmond U., Salcedo R., Alexopoulou L., Stoye J.P., Kassiotis G. (2012). Resurrection of endogenous retroviruses in antibody-deficient mice. Nature.

[66] Zhou F., Li M., Wei Y., Lin K., Lu Y., Shen J. (2016). Activation of HERV-K Env protein is essential for tumorigenesis and metastasis of breast cancer cells. Oncotarget.

[67] Trela M., Nelson P.N., Rylance P.B. (2016). The role of molecular mimicry and other factors in the association of Human Endogenous Retroviruses and autoimmunity. APMIS.

